# Steroid vs. antibiotic impregnated absorbable nasal packing for wound healing after endoscopic sinus surgery: a randomized, double blind, placebo-controlled study^☆^

**DOI:** 10.1016/j.bjorl.2018.04.002

**Published:** 2018-05-10

**Authors:** Blazej Grzeskowiak, Malgorzata Wierzchowska, Rafal Walorek, Malgorzata Seredyka-Burduk, Katarzyna Wawrzyniak, Pawel Krzysztof Burduk

**Affiliations:** a University Hospital, Department of Otolaryngology and Laryngological Oncology, Bydgoszcz, Poland; b Nicolaus Copernicus University, Faculty of Medicine, Department of Otolaryngology and Laryngological Oncology, Collegium Medicum, Toruń, Poland; c Nicolaus Copernicus University, Faculty of Medicine, Department of Optometry, Collegium Medicum, Toruń, Poland; d Nicolaus Copernicus University, Faculty of Medicine, Department of Anesthesiology and Intensive Therapy, Collegium Medicum, Toruń, Poland

**Keywords:** Anti-bacterial agents, Steroids, Wound healing, Endoscopic sinus surgery, Nasopore/biodegradable packing, Agentes antibacterianos, Esteroides, Cicatrização de feridas, Cirurgia endoscópica nasal, Nasopore/tampão biodegradável

## Abstract

**Introduction:**

Endoscopic sinus surgery can lead to crusting or synechiae formation, which can affect the healing process.

**Objective:**

The aim of our study was to compare the influence of steroid versus antibiotic versus saline solution impregnated absorbable nasal spacers on postoperative wound healing and patient satisfaction.

**Methods:**

Eighty patients, 33 women and 47 men, were enrolled in this study. At the end of the surgery, two pieces of 4 cm biodegradable material were applied in each ethmoid cavity. One of them was impregnated with saline solution, while the second one with steroid, or with antibiotic.

**Results:**

We observed statistically significant differences in the Lund–Kennedy score between the control and both treatment groups: for the Antibiotic-group on days 10 and 30 (*p* = 0.009; *p* = 0.009) and for the Steroid-group on day 90 (*p* = 0.008). The extended endoscopic appearance of nasal mucosa indicated statistically significant differences in crust formation on day 10 comparing the steroid and control dressing (*p* = 0.025), in secretion type on days 10 and 30 comparing the antibiotic and control dressing (*p* = 0.003; *p* = 0.016) and additionally for steroid and control on day 90 (*p* = 0.046). On Day 90 we observed statistically significant differences in the absence of mucosal edema in the S-group compared to controls (*p* = 0.007).

**Conclusions:**

The results of this study reveal the significant positive influence of steroid- and antibiotic-impregnated biodegradable nasal packing on the postoperative healing process and patient satisfaction compared to the saline soaked dressing.

## Resumo

**Introdução:**

A cirurgia endoscópica nasossinusal pode levar à formação de crostas e sinéquias, o que pode afetar o processo de cicatrização.

**Objetivo:**

O objetivo do nosso estudo foi comparar a influência do espaçador nasal absorvível embebido em esteroide versus antibiótico versus solução salina na cicatrização de ferida pós-operatória e na satisfação do paciente.

**Método:**

Oitenta pacientes, 33 mulheres e 47 homens, foram incluídos neste estudo. Ao final da cirurgia, dois tampões de material biodegradável de 4 cm foram aplicados em cada cavidade etmoidal. Um deles foi embebido em solução salina, enquanto no segundo foi utilizado esteroide, ou antibiótico.

**Resultados:**

Observamos diferenças estatisticamente significantes no escore de Lund-Kennedy entre os grupos controle e ambos os grupos tratamentos: para o grupo antibiótico nos dias 10 e 30 (*p* = 0,009; *p* = 0,009) e para o grupo esteroide no dia 90 (*p* = 0,008). O aspecto endoscópico da mucosa nasal indicou diferenças estatisticamente significantes na formação de crostas no dia 10, na comparação do esteroide com o curativo controle (*p* = 0,025), no tipo de secreção nos dias 10 e 30, na comparação do antibiótico com o curativo controle (*p* = 0,003; *p* = 0,016) e adicionalmente para esteroide e controle no dia 90 (*p* = 0,046). No dia 90, observamos diferenças estatisticamente significantes na ausência de edema da mucosa no grupo E (esteroide) em relação aos controles (*p* = 0,007).

**Conclusões:**

Os resultados deste estudo revelam uma influência positiva significante no uso de tampão nasal biodegradável embebido em esteroides e antibióticos no processo de cicatrização pós-operatória e satisfação do paciente em comparação com o curativo embebido em solução salina.

## Introduction

Endoscopic sinus surgery (ESS), which allows topical medication to enter the nasal and paranasal cavities to treat the mucosa,^1^ is the preferred method of surgery for chronic rhinosinusitis (CRS). The relatively high rate (20–26%), of revision surgery due to recurrent disease necessitates the re-evaluation of the optimal postoperative treatment.^1^ The main thing responsible for successful outcomes after ESS is proper and ongoing wound healing. Factors that facilitate poor results are crusting or synechiae formation between the middle turbinate and lateral nasal wall cause lateralization of the turbinate and block the opened sinuses.^2^, ^3^ On the other hand infection or persistent local inflammation in the operated sinus spaces could prolong the healing process and lead to surgical failure. There are several possibilities of postoperative care to prevent crust or synechiae formation, but none of them is highly successful. Absorbable and non-absorbable materials are used as middle meatal spacers, but it is still debatable which of them is better.^2^, ^4^ The absorbable material improves patients comfort as it dissolves and can be washed out or, in some cases, suctioned out.^4^, ^5^ It is very important to recommend to patients, when using absorbable materials, to irrigate the nose and sinuses multiple times per day. This is obligatory in the first days after the surgery, otherwise some remnants of dressing would slow down the healing process. Moreover, partial dissolution of the material may be the cause of crusting, synechiae formation or even influence mucosal regeneration by activating osteogenesis.^4^, ^5^ Using topical anti-bacterial agents or steroids could effectively influence the postoperative outcome by reduction of inflammation and nasal secretion.^6^ Recently, it has been seen that absorbable material impregnated with drugs, mostly steroids, could be a promising method for avoiding crusting and synechiae formation.^2^, ^6^, ^7^, ^8^, ^9^

We conducted a prospective randomized study in CRS patients to find out whether the choice of a steroid versus antibiotic versus saline solution-impregnated absorbable nasal spacer (Nasopore, Stryker) has a potential effect on postoperative wound healing and patient satisfaction following ESS.

## Methods

### Patients

A total of 80 adult patients with CRS, with and without nasal polyps, who underwent bilateral ESS, were recruited for the study. In the study group, 11 patients were diagnosed with asthma (5 in the antibiotic group and 6 in the steroid group) and one case of Aspirin Exacerbated Respiratory Disease (AERD) in each group. The diagnosis of CRS was based on history, clinical endoscopic examination and CT of the paranasal sinuses. The exclusion criteria were: rhinosinusitis limited to only one sinus or one side; known intolerance to polyurethane or bethametasone or ciprofloxacin, glaucoma, sinonasal neoplasia and pregnancy. Patients with a clinical history of facial trauma or those with complete removal of the middle turbinate secondary to surgery or rhinoplasty were also excluded. Details of the study groups are presented in Table 1.

**Table 1:** Baseline characteristic.

		Group		Total
		Antibiotic	Steroid	
*Gender*				
Females	*n*	18	15	33
	%	45.0	37.5	41.25
Males	*n*	22	25	47
	%	55.0	62.5	58.75

*Total*		40	40	80

*Age (years)*				
Mean		42.95	47.73	45.34
SD		14.422	12.447	13.435

The research project was approved by the local Ethics Committee no KB 326/2013, and written informed consent was obtained before the study.

### Study design

This was a prospective, randomized double-blinded and placebo-controlled study comparing the effect of steroid-impregnated (S group) vs. antibiotic-impregnated (A group) to saline solution-impregnated (control group) biodegradable (Nasopore, Stryker) packing used after ESS, with regard to subjective symptoms and postoperative wound healing in short and long term follow-up. All the patients underwent bilateral complete ethmoidectomy, middle meatal antrostomy and sphenoidectomy or frontal recess surgery if necessary. ESS was performed by one surgeon. The set of 80 envelopes including the type of drug used and application side was prepared, then random numbers generated online using Research Randomizer (www.randomizer.org) were given to the envelopes. At the end of the surgery two pieces of 4 cm biodegradable material (Nasopore) were applied in each ethmoid cavity. For the blinding, the OR nurse prepared adequate solutions and soaked the dressing, according to the instructions in the envelope. From this moment, the instructions stay in the closed envelope to the end of the trial. The patients from the S group received 2 mL with 3.5 mg of Betamethazon (Diprophos, MSD Poland) on one side, while patients from the A group received 2 mL with 5 mg of Ciprofloxacin (Ciprinol, KRKA Poland). On the control side, the dressing was impregnated with 2 mL of saline solution in both groups. Patients were instructed to irrigate the nose with saline 4 times a day and to use intranasal topical steroids starting from 2 weeks after the surgery. A postoperative systemic antibiotic (Ciprofloxacin 500 mg twice) was administrated orally to all patients for 2 weeks.

### Assessment of outcomes

All the patients underwent CT scans and were scored using the Lund–Mackay grading system.^1^, ^10^ Subjective symptoms: headache, nasal pain, pressure, nose blockage, itching, nose bleeding as well as smell were assessed postoperatively on postoperative day 10, and at 1, 3, and 6 months using the VAS scale. The healing process was assessed by recording for edema, crusting, secretion and scarring on postoperative day 10, and at 1, 3 and 6 months using the validated Lund–Kennedy and Postoperative Sinus Endoscopy (POSE) scores by one person for all cases observed.^6^, ^11^ The post-operative control visits were performed in the absence of the operating surgeon.

### Statistical analysis

All statistical analyses were performed using Statistica software, v. 10 (StatSoft Inc.). The normal distribution was checked with Kolmogorov–Smirnov test. Paired test were done with Mann–Whitney test and for unpaired Kruskal–Wallis test were done with significance levels set at *p* < 0.05. The study population (40 for each group) was calculated for error inherent in a test result. The power analysis of the study was 80%.

## Results

Eighty patients were included in this study. The mean age in A group was 42.95 years, and in the S group it was appropriately 47.73 years. The sex distribution was comparable in both study groups (Table 1). The mean preoperative Lund–Mackay CT score on the tested sides for the A group was 8.45 ± 3.218, while the score for the S group was 7.50 ± 2.699, and was comparable to the control group scores: 8.63 ± 3.044 and 7.45 ± 2.810.

**Figure 1:**
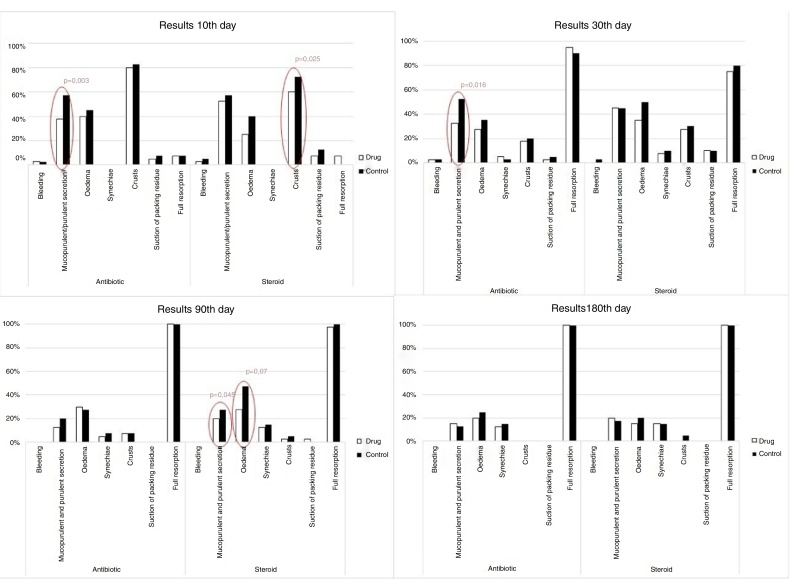
Endoscopic appearance of nasal mucosa – steroid, antibiotic and control group.

The objective endoscopic scoring system for sinonasal cavities (Lund–Kennedy) and the endoscopic appearance of nasal mucosa was used to follow-up the healing process (Table 2, Table 3) (Fig. 1).

**Table 2:** Lund–Kennedy post operation score.

Day	Antibiotic (*n* = 40)			*p* between groups	Steroid (*n* = 40)		
	Drug	Control	*p*		Drug	Control	*p*
Baseline	4.00 ± 1.396	4.08 ± 1.269	0.66	0.297	3.40 ± 1.257	3.45 ± 1.197	0.623
10	0.67 ± 0.772	1.05 ± 0.826	0.009	0.633	0.68 ± 0.797	0.95 ± 0.986	0.087
30	0.64 ± 0.811	0.85 ± 0.812	0.009	0.051	1.00 ± 1.076	1.28 ± 1.169	0.096
90	0.57 ± 0.867	0.62 ± 0.953	0.527	0.321	0.58 ± 0.889	0.97 ± 1.219	0.008
180	0.54 ± 1.039	0.60 ± 1.117	0.157	0.075	0.56 ± 1.021	0.76 ± 1.458	0.084

Mean ± SD; Wilcoxon signed rank test for pairs; Kruskal–Wallis test between group.

**Table 3:** Post-operative endoscopic score.

Group	Parameter	Scale	Packing type	10 day (Mean ± SD)	30 day (Mean ± SD)	3 month (Mean ± SD)	6 month (Mean ± SD)
Antibiotic	Bleeding	0–2	Drug	0.00	0.03 ± 0.160	0.00	0.00
			Control	0.00	0.00 ± 0.000	0.00	0.00
			*p*	1.000	0.317	1.000	1.000
	Secretion	0–3	Drug	0.41 ± 0.549	0.33 ± 0.478	0.14 ± 0.347	0.17 ± 0.382
			Control	0.72 ± 0.686	0.59 ± 0.595	0.22 ± 0.417	0.14 ± 0.355
			*p*	0.003	0.016	0.257	0.317
	Oedema	0–3	Drug	0.41 ± 0.498	0.33 ± 0.577	0.46 ± 0.730	0.49 ± 0.981
			Control	0.46 ± 0.505	0.36 ± 0.486	0.46 ± 0.767	0.60 ± 1.035
			*p*	0.527	0.683	1.000	0.102
	Crusting	0–1	Drug	0.82 ± 0.389	0.18 ± 0.389	0.00	0.00
			Control	0.85 ± 0.366	0.21 ± 0.409	0.00	0.00
			*p*	0.317	1.000	1.000	1.000
	Synechiae		Drug	0.00	0.05 ± 0.223	0.03 ± 0.164	0.14 ± 0.355
			Control	0.00	0.02 ± 0.160	0.00	0.17 ± 0.382
			*p*	1.000	1.000	0.317	0.655
Steroid	Bleeding	0–2	Drug	0.03 ± 0.158	0.00	0.00	0.00
			Control	0.05 ± 0.221	0.00	0.00	0.00
			*p*	0.317	1.000	1.000	1.000
	Secretion	0–3	Drug	0.55 ± 0.552	0.62 ± 0.781	0.21 ± 0.413	0.26 ± 0.511
			Control	0.65 ± 0.622	0.54 ± 0.642	0.32 ± 0.525	0.24 ± 0.496
			*p*	0.286	0.724	0.046	0.655
	Oedema	0–3	Drug	0.25 ± 0.439	0.46 ± 0.682	0.37 ± 0.633	0.32 ± 0.727
			Control	0.40 ± 0.496	0.67 ± 0.737	0.68 ± 0.775	0.44 ± 0.849
			*p*	0.083	0.149	0.007	0.157
	Crusting	0–1	Drug	0.60 ± 0.496	0.28 ± 0.456	0.00	0.00
			Control	0.73 ± 0.452	0.31 ± 0.468	0.00	0.00
			*p*	0.025	1.000	1.000	1.000
	Synechiae		Drug	0.00	0.13 ± 0.409	0.13 ± 0.343	0.21 ± 0.479
			Control	0.00	0.18 ± 0.451	0.16 ± 0.370	0.24 ± 0.554
			*p*	1.000	0.617	0.564	0.705

Statistically significant differences in the Lund–Kennedy scores were observed for the treatment and control groups, respectively: for A group on days 10 and 30 (*p* = 0.009, *p* = 0.009), and for the S group on day 90 (*p* = 0.008). Differences, but not statistically significant, were observed between treatment groups, although on day 30 the differences were most evident (*p* = 0.051) (Table 2).

The endoscopic appearance of nasal mucosa showed statistically significant differences in crust formation on day 10 compared to the S group and the control group (*p* = 0.025) (Table 3) (Fig. 1). On day 90 we observed statistically significant differences in the absence of mucosal edema in the S group compared to controls (*p* = 0.007). The reduction of edema for the S group was seen at all times of the experiment, but it achieved the statistical threshold only on day 90 (Table 4) (Fig. 1). There were also statistically significant differences in the type of secretion in the A group on days 10 and 30 (*p* = 0.003; *p* = 0.016), and also in the S group on day 90 (*p* = 0.046). No statistically significant differences were found between the groups.

**Table 4:** Post-operative symptoms rating in VAS scale.

Group	Symptom	Packing type	Day									
			2		10		30		90		180	
			Mean ± SD	*p*	Mean ± SD	*p*	Mean ± SD	*p*	Mean ± SD	*p*	Mean ± SD	*p*
Antibiotic	Facial pressure	Drug	1.65 ± 2.020	0.050	0.51 ± 1.189	0.011	0.38 ± 0.963	0.066	0.24 ± 0.760	0.048	0.54 ± 1.597	0.317
		Control	1.88 ± 2.300		0.90 ± 1.569		0.90 ± 1.774		0.57 ± 1.042		0.57 ± 1.632	
	Nasal occlusion	Drug	3.45 ± 2.012	0.200	1.67 ± 1.722	0.356	0.95 ± 1.905	0.032	0.32 ± 0.784	0.000	0.54 ± 1.400	0.041
		Control	1.40 ± 2.036		1.92 ± 1.723		1.46 ± 1.862		1.00 ± 1.269		0.83 ± 1.807	
	Smell	Drug	2.60 ± 3.103	0.007	5.28 ± 3.077	0.008	7.00 ± 3.656	0.039	8.08 ± 3.303	0.102	8.34 ± 3.378	0.317
		Control	0.48 ± 1.012		4.92 ± 3.157		6.69 ± 3.599		7.95 ± 3.33		8.32 ± 3.411	
Steroid	Facial pressure	Drug	1.25 ± 1.532	0.028	1.33 ± 2.043	0.329	1.00 ± 1.622	0.822	0.47 ± 1.179	0.102	0.29 ± 0.906	0.317
		Control	1.73 ± 2.207		1.53 ± 2.160		1.00 ± 1.338		0.66 ± 1.512		0.35 ± 1.070	
	Nasal occlusion	Drug	3.13 ± 2.430	0.221	1.33 ± 1.730	0.098	1.15 ± 1.927	0.596	0.62 ± 1.010	0.033	0.38 ± 1.704	1.000
		Control	3.43 ± 2.630		1.80 ± 1.829		1.31 ± 1.908		1.16 ± 1.653		0.38 ± 0.954	
	Smell	Drug	3.85 ± 3.958	0.258	6.50 ± 3.404	0.246	7.56 ± 3.033	0.593	8.55 ± 2.806	0.785	9.47 ± 1.461	0.317

The VAS scale results for headache, nasal pain, pressure, nose blockage; itching, nose bleeding and smell were recorded. Among all those complaints statistically significant differences were found in facial pressure, nasal blockage and smell. A difference concerning facial pressure was found in the A group throughout the observation time, but a statistical threshold was achieved on days 10 and 90 (*p* = 0.011; *p* = 0.048). Statistically less pressure was also reported on day 10 in the S group (*p* = 0.028). Nasal blockage was significantly diminished in the A group on days 30, 90 and 180 (*p* = 0.032; *p* = 0.000; *p* = 0.041) and in the S group on day 90 (*p* = 0.033). Statistically better smell was reported for the A group compared to placebo group on days 2, 10 and 30 (*p* = 0.007; *p* = 0.008; *p* = 0.039, respectively).

Pressure and nasal blockage were less observed in the A-group and in the S group, respectively, for pressure on day 10 (*p* < 0.01) and for nose blockage on day 90 (*p* < 0.01) when compared to antibiotic vs. control. The differences between the A group and S group did not achieve the statistical threshold in any of the investigated parameters.

## Discussion

The most important parameters of the healing process after ESS are reduction of scarring, edema, crusting and lateralization of the middle turbinate. On the other hand, the benefits of nasal packing could be diminished due to patients’ discomfort in terms of headache, nasal pain, pressure, or nasal blockage.^4^, ^12^, ^13^, ^14^ Many biodegradable materials have been developed and are used as middle meatal spacers after ESS.^4^, ^15^, ^16^, ^17^ The previous studies, however, reported variable results. Shoman et al. observed that Nasopore (synthetic polyurethane) does not reduce discomfort compared to Merocel, but could also slow down the healing process in the early follow-up period.^5^ The same results were obtained by Burduk et al.^4^ This is probably due to incomplete resorption of the material resulting from patients avoiding intense nasal irrigation. The remnants of the sponge (NasoPore) could form bridges, increased osteogenesis and synechiae formation. This was observed in a previous study.^4^, ^5^ In some studies, the absorbable material was impregnated with topical steroids to reduce synechiae formation.^2^, ^6^, ^9^ The topical steroid acts as an anti-inflammatory drug and moderates the healing process. On the other hand, the use of a topical antibiotic for nasal packing could prevent infections and, as a consequence of this influence on the healing process, reduce crusting and synechiae formation.^18^ We also observed better dissolvable parameters, although not statistically significant, when the NasoPore was soaked with antibiotic and steroid rather than the saline solution.

In our prospective, double-blinded, randomized controlled study we compared the efficacy of wound healing and patient satisfaction depending on the application of biodegradable packing (Nasopore) impregnated with a topical steroid or an antibiotic. Ciprofloxacin was used because of its broad antimicrobial spectrum, easy accessibility in ENT operating rooms, and safety of topical application. Moreover, at the starting point of the trial, fluoroquinolones were included in the Polish recommendations for alternative antibiotic therapy in rhinosinusitis. Rejection of the initial empirical antimicrobial drugs group was done due to its abuse in patients with chronic rhinosinusitis. Betamethasone was used because of its strong anti-inflammatory effect and safety of topical application.

**Figure 2:**
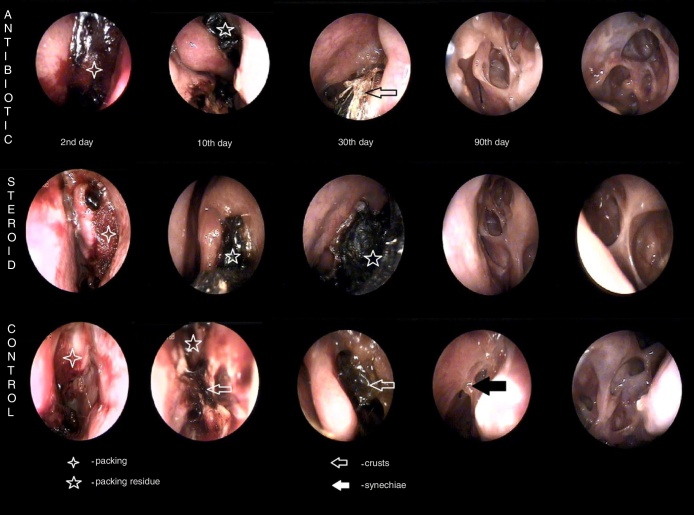
Endoscopic follow-up resorption and healing process between groups.

The steroid-impregnated biodegradable nasal packing group compared to the control group had a better outcome in the reduction of mucosal edema at each point of the follow-up. We also observed a lower frequency of pathological secretions (Lund–Kennedy score). Although all of the patients used a systemic antibiotic after the surgery, we did not observe any influence of it on crusting or secretion in the early post-operative follow-up. In the early post-operative period the use of a steroid was responsible for sporadic episodes of bleeding, up to 10 days, and statistically significant reduction of crust. In some cases we observed a delay in Nasopore resorption, which could probably be a reason for headache up to 30 days post operation (Figure 1, Figure 2).

The same results were obtained by other authors, and indicated the improvement in the healing process with reduction of oedema, crusting and scarring.^2^, ^4^, ^6^, ^19^ In the group of nasal packing impregnated with a topical antibiotic, in comparison to the control group, we also observed a better outcome of the healing process. Up to 30 days postoperatively we recorded decreased mucous and purulent secretion. The subjective symptoms of patients’ comfort and satisfaction were more expressed. We documented a decrease in the feeling of nose blockage, decreased facial pressure, and improved smell. Better healing after ESS when using a topical antibiotic was also observed by Snikani.^18^ The author described less crusting and incidence of bacterial infection, as well as reduction or elimination of pain when removing nonabsorbable packing.

In the present study we also compared the benefits of the healing process and patient satisfaction when using steroid- or antibiotic-impregnated biodegradable nasal packing. To our knowledge this is the first comparison of two topical drugs used after ESS. We observed no statistically significant differences in each of the investigated parameters. This could probably be due to the better sensitivity of the paired statistical test used for each group in comparison to the unpaired tests used between groups. The ideal single or multiple drug regimen to achieve the best clinical results after ESS remain to be clarified. Our study demonstrated that both topical drugs have potential influence on the healing process and final clinical outcome. Future studies are needed to determine the ideal dosing of a single drug, or mixture of drugs, to achieve the best clinical results after ESS.

## Conclusions

The results of this study reveal a significant improvement of steroid and antibiotic impregnated biodegradable nasal packing influence on the postoperative healing process compared to saline soaked dressing. However, antibiotic-impregnated packing demonstrated better advantage over the steroid dressing with regard to patient comfort and satisfaction. Future investigations should be focused on the combination of both active drugs administered simultaneously in the ethmoid spaces.

## Conflicts of interest

The authors declare no conflicts of interest.
